# Correction: Using Qualitative Evidence in Decision Making for Health and Social Interventions: An Approach to Assess Confidence in Findings from Qualitative Evidence Syntheses (GRADE-CERQual)

**DOI:** 10.1371/journal.pmed.1002065

**Published:** 2016-06-10

**Authors:** The PLOS Medicine Staff

## Notice of Republication

This article was republished on May 18, 2016, to fix an issue with broken supporting information hyperlinks that was introduced during the typesetting process. The publisher apologizes for the errors. Please download this article again to view the correct version. The originally published, uncorrected article and the republished, corrected articles are provided here for reference.

## Supporting Information

S1 File Originally published, uncorrected article. (PDF)

S2 File Republished, corrected article. (PDF)
