# Supplementary material for: Using Qualitative Evidence in Decision Making for Health and Social Interventions: An Approach to Assess Confidence in Findings from Qualitative Evidence Syntheses (GRADE-CERQual)
Source: PLoS Med. 2015 Oct 27;12(10):e1001895. doi: 10.1371/journal.pmed.1001895 (PMC4624425; doi:10.1371/journal.pmed.1001895)
Supplement: S1 Table — (PDF) [file pmed.1001895.s001.pdf]

**Supplementary table 1: Key definitions relevant to CERQual**

| |
|---|
| **Qualitative evidence synthesis:** A systematic review of primary qualitative studies.<br><br>**Review question:** The overall question addressed by a qualitative evidence synthesis. Typically the review question specifies the perspective or population, the phenomenon of interest or intervention, and the setting to be examined in the synthesis.<br><br>**Phenomenon of interest:** The issue that is the focus of qualitative inquiry. It is often embedded within the review question. The phenomenon of interest may be a health or social intervention, such as a new vaccine or lunch clubs for the elderly, or a health or social issue, such as living with HIV/AIDS or being on welfare.<br><br>**Review finding**: An analytic output from a qualitative evidence synthesis that describes the phenomenon of interest or an aspect of a phenomenon. Review findings are based on data from included qualitative studies. A qualitative evidence synthesis typically includes a number of review findings.<br><br>**Data:** Information obtained from individual qualitative research studies including quotes from participants and/or researcher metaphors, concepts and interpretations.<br><br>**Confidence:** Our confidence in the evidence is an assessment of the extent to which the review finding is a reasonable representation of the phenomenon of interest. That is, the phenomenon of interest is unlikely to be substantially different from the research finding. By substantially different, we mean different enough that it might change how the finding influences a practical or policy decision about a health, social care or other intervention.<br><br>**CERQual components**: The four CERQual components represent issues to consider when assessing how much confidence to place in the findings from qualitative evidence syntheses. The four components are methodological limitations, relevance, coherence and adequacy of data. Each component is assessed with respect to an individual review finding, the evidence supporting that review finding, and how the review finding relates to the wider review question. |
