# Supplementary material for: Using Qualitative Evidence in Decision Making for Health and Social Interventions: An Approach to Assess Confidence in Findings from Qualitative Evidence Syntheses (GRADE-CERQual)
Source: PLoS Med. 2015 Oct 27;12(10):e1001895. doi: 10.1371/journal.pmed.1001895 (PMC4624425; doi:10.1371/journal.pmed.1001895)
Supplement: S2 Table — (PDF) [file pmed.1001895.s002.pdf]

Supplementary table 2: Comparison of the CERQual components and the elements of GRADE

**CERQual components for assessing confidence in the evidence for findings from syntheses of qualitative evidence**	**GRADE elements for assessing the certainty of evidence, by outcome, on the effectiveness of interventions** [1]
Methodological limitations	Limitations in study design / risk of bias
Relevance	Indirectness
Coherence	Inconsistency
Adequacy of data	Imprecision
*Dissemination bias**	Publication bias

* The inclusion of dissemination bias in CERQual is still under discussion – see Boxes 2 and 4

References

1. Balshem H, Helfand M, Schunemann HJ, Oxman AD, Kunz R, et al. (2011) GRADE guidelines: 3. Rating the quality of evidence. J Clin Epidemiol 64: 401-406.
