# Supplementary material for: Using Qualitative Evidence in Decision Making for Health and Social Interventions: An Approach to Assess Confidence in Findings from Qualitative Evidence Syntheses (GRADE-CERQual)
Source: PLoS Med. 2015 Oct 27;12(10):e1001895. doi: 10.1371/journal.pmed.1001895 (PMC4624425; doi:10.1371/journal.pmed.1001895)
Supplement: S1 Alternative Language Summary Points — French translation of the Summary Points. (PDF) [file pmed.1001895.s003.pdf]

Résumé en points clés

- Les synthèses d’études qualitatives sont de plus en plus utilisées, cependant les méthodes qui permettent d’apprécier le niveau de confiance à accorder aux résultats de ces synthèses sont peu développées.
- L’approche « Confiance aux données provenant des synthèses d’études qualitatives » (CERQual) permet d’apprécier le niveau de confiance à accorder aux résultats d’une synthèse qualitative.
- L’évaluation de la confiance CERQual dans chaque résultat d’une synthèse qualitative se base sur quatre composantes : les limites méthodologiques des études qualitatives qui contribuent au résultat, la pertinence de la question de recherche des études qui contribuent à un résultat, la cohérence du résultat, et l'adéquation des données à l'appui d'un résultat.
- CERQual fournit une méthode transparente pour évaluer la confiance dans les résultats des synthèses qualitatives. Comme dans GRADE pour les preuves sur l'efficacité, l’approche CERQual peut faciliter l'utilisation de données qualitatives pour éclairer les décisions et orienter les politiques.
- L'approche CERQual est en cours de développement par un sous-groupe du Groupe de travail GRADE.
