# Supplementary material for: Using Qualitative Evidence in Decision Making for Health and Social Interventions: An Approach to Assess Confidence in Findings from Qualitative Evidence Syntheses (GRADE-CERQual)
Source: PLoS Med. 2015 Oct 27;12(10):e1001895. doi: 10.1371/journal.pmed.1001895 (PMC4624425; doi:10.1371/journal.pmed.1001895)
Supplement: S2 Alternative Language Summary Points — Italian translation of the Summary Points. (PDF) [file pmed.1001895.s004.pdf]

Punti essenziali

- Le sintesi di evidenze qualitative sono sempre più utilizzate, sebbene i metodi per determinare quanta affidabilità dare ai risultati ottenuti sono poco sviluppati.
- L'approccio Confidence in the Evidence from Reviews of Qualitative research (CERQual) aiuta a valutare quanta affidabilità assegnare ai risultati a partire da una sintesi di evidenze qualitative.
- La valutazione di affidabilità di CERQual per i risultati individuali di una revisione a partire dalla sintesi di evidenze qualitative si basa su quattro componenti: le limitazioni metodologiche degli studi qualitativi che contribuiscono ai risultati di una revisione, la rilevanza rispetto alla domanda della revisione degli studi che contribuiscono ai risultati di una revisione, la coerenza dei risultati della revision, e l'adeguatezza dei dati che supportano I risultati di una revisione.
- CERQual fornisce un metodo trasparente per valutare l'affidabilità dei risultati della sintesi delle evidenze qualitative. Come per l'approccio Grading of Recommendations Assessment, Development, and Evaluation (GRADE) per l'evidenza dell'efficacia, CERQual può facilitare l'uso delle evidenze qualitative per informare le decisioni e definire le politiche.
- L'approccio CERQual viene sviluppato da un sottogruppo del gruppo di lavoro GRADE.
