# Supplementary material for: Using Qualitative Evidence in Decision Making for Health and Social Interventions: An Approach to Assess Confidence in Findings from Qualitative Evidence Syntheses (GRADE-CERQual)
Source: PLoS Med. 2015 Oct 27;12(10):e1001895. doi: 10.1371/journal.pmed.1001895 (PMC4624425; doi:10.1371/journal.pmed.1001895)
Supplement: S3 Alternative Language Summary Points — Norwegian translation of the Summary Points. (PDF) [file pmed.1001895.s005.pdf]

Oppsummering

- Systematiske oversikter av kvalitativ forskning brukes i økende grad, men metoder for å vurdere hvor mye tillit vi har til resultatene fra slike oversikter er lite utviklet.
- CERQual-metoden («Confidence in the Evidence from Reviews of Qualitative research») hjelper oss å vurdere hvor mye tillit vi bør ha til resultater fra systematiske oversikter av kvalitativ forskning.
- Vurderingen ved hjelp av CERQual hviler på en vurdering av fire komponenter: de metodologiske begrensningene ved de kvalitative studiene som har bidratt til hvert funn, relevansen av studiene som har bidratt til hvert funn, datasamsvar på tvers av studiene, og omfanget av dataene som støtter opp under hvert funn.
- CERQual er en gjennomsiktig metode for vurdering av tillit til resultater fra systematiske oversikter av kvalitativ forskning. I likhet med GRADE-tilnærmingen («Grading of Recommendations Assessment, Development, and Evaluation»), som brukes for effektforskning, kan CERQual legge til rette for bruk av kvalitativ forskning i politikkutforming og andre beslutningsprosesser.
- CERQual-metoden blir utviklet av en undergruppe av GRADE Working Group.
