# Supplementary material for: Using Qualitative Evidence in Decision Making for Health and Social Interventions: An Approach to Assess Confidence in Findings from Qualitative Evidence Syntheses (GRADE-CERQual)
Source: PLoS Med. 2015 Oct 27;12(10):e1001895. doi: 10.1371/journal.pmed.1001895 (PMC4624425; doi:10.1371/journal.pmed.1001895)
Supplement: S4 Alternative Language Summary Points — Spanish translation of the Summary Points. (PDF) [file pmed.1001895.s006.pdf]

Puntos clave

- Las síntesis de evidencia cualitativa se utilizan cada vez más, pero los métodos para evaluar el grado de confianza que podemos depositar en los hallazgos de estas síntesis están poco desarrollados.
- La propuesta CERQual (*Confidence in the Evidence from Reviews of Qualitative research*) es una herramienta que ayuda a evaluar la confianza que podemos depositar en los hallazgos de una síntesis de evidencia cualitativa.
- La evaluación de la confianza en hallazgos provenientes de revisiones de síntesis de evidencia cualitativa con CERQual se basa en cuatro componentes: las limitaciones metodológicas de los estudios cualitativos que contribuyen a los hallazgos de la revisión, la relevancia de los estudios contribuyentes a para los resultados y la pregunta de la revisión, la coherencia de los hallazgos de la revisión, y la adecuación de los datos que sustentan el hallazgo de una revisión.
- CERQual ofrece un método transparente para evaluar el grado de confianza en los hallazgos de las síntesis cualitativas. Al igual que la propuesta GRADE (Grading of Recommendations Assessment, Development, and Evaluation) para pruebas de efectividad, CERQual puede facilitar el uso de la evidencia cualitativa para informar las decisiones y determinar políticas.
- La propuesta CERQual está siendo desarrollado por un subgrupo del grupo de trabajo GRADE.
