# Supplementary material for: Correction: Using Qualitative Evidence in Decision Making for Health and Social Interventions: An Approach to Assess Confidence in Findings from Qualitative Evidence Syntheses (GRADE-CERQual)
Source: PLoS Med. 2016 Jun 10;13(6):e1002065. doi: 10.1371/journal.pmed.1002065 (PMC4902189; doi:10.1371/journal.pmed.1002065)
Supplement: S1 File — (PDF) [file pmed.1002065.s001.PDF]

PLOS | MEDICINE

GUIDELINES AND GUIDANCE

Using Qualitative Evidence in Decision Making for Health and Social Interventions: An Approach to Assess Confidence in Findings from Qualitative Evidence Syntheses (GRADE-CERQual)

Simon Lewin[1,2]*, Claire Glenton[1], Heather Munthe-Kaas[3], Benedicte Carlsen[4], Christopher J. Colvin[5], Metin Gülmezoglu[6], Jane Noyes[7], Andrew Booth[8], Ruth Garside[9], Arash Rashidian[10,11]

1 Global Health Unit, Norwegian Knowledge Centre for the Health Services, Oslo, Norway, **2** Health Systems Research Unit, South African Medical Research Council, Cape Town, South Africa, **3** Social Welfare Unit, Norwegian Knowledge Centre for the Health Services, Oslo, Norway, **4** Uni Research Rokkan Centre, Bergen, Norway, **5** Division of Social and Behavioural Sciences, School of Public Health and Family Medicine, University of Cape Town, Cape Town, South Africa, **6** UNDP/UNFPA/ UNICEF/WHO/World Bank Special Programme of Research, Development and Research Training in Human Reproduction, Department of Reproductive Health and Research, WHO, Geneva, Switzerland, **7** School of Social Sciences, Bangor University, Bangor, United Kingdom, **8** School of Health & Related Research (ScHARR), University of Sheffield, Sheffield, United Kingdom, **9** European Centre for Environment and Human Health, University of Exeter Medical School, Exeter, United Kingdom, **10** Department of Health Management and Economics, School of Public Health, Tehran University of Medical Sciences, Tehran, Iran, **11** Department of Information, Evidence and Research, Eastern Mediterranean Region, World Health Organization, Cairo, Egypt

* simon.lewin@nokc.no

CrossMark
click for updates

OPEN ACCESS

Citation: Lewin S, Glenton C, Munthe-Kaas H, Carlsen B, Colvin CJ, Gülmezoglu M, et al. (2015) Using Qualitative Evidence in Decision Making for Health and Social Interventions: An Approach to Assess Confidence in Findings from Qualitative Evidence Syntheses (GRADE-CERQual). PLoS Med 12(10): e1001895. doi:10.1371/journal.pmed.1001895

Published: October 27, 2015

Copyright: © 2015 Lewin et al. This is an open access article distributed under the terms of the Creative Commons Attribution License, which permits unrestricted use, distribution, and reproduction in any medium, provided the original author and source are credited.

Funding: This work was supported by funding from the Department of Reproductive Health and Research, WHO (www.who.int/reproductivehealth/about_us/en/) and Norad (Norwegian Agency for Development Cooperation: www.norad.no) to the Norwegian Knowledge Centre for the Health Services. Additional funding for several of the pilot reviews was provided by the Alliance for Health Policy and Systems Research (www.who.int/alliance-hpsr/en/). We also received funding for elements of this work through the Cochrane supported "Methodological Investigation of Cochrane reviews of Complex Interventions" (MICCI) project (www.cochrane.org). SL is supported by funding from the South African Medical Research Council (www.mrc.ac.za). The funders had no role in study design, data

Summary Points

- Qualitative evidence syntheses are increasingly used, but methods to assess how much confidence to place in synthesis findings are poorly developed.
- The Confidence in the Evidence from Reviews of Qualitative research (CERQual) approach helps assess how much confidence to place in findings from a qualitative evidence synthesis.
- CERQual's assessment of confidence for individual review findings from qualitative evidence syntheses is based on four components: the methodological limitations of the qualitative studies contributing to a review finding, the relevance to the review question of the studies contributing to a review finding, the coherence of the review finding, and the adequacy of data supporting a review finding.
- CERQual provides a transparent method for assessing confidence in qualitative evidence syntheses findings. Like the Grading of Recommendations Assessment, Development, and Evaluation (GRADE) approach for evidence of effectiveness, CERQual may facilitate the use of qualitative evidence to inform decisions and shape policies.
- The CERQual approach is being developed by a subgroup of the GRADE Working Group.

collection and analysis, preparation of the manuscript or the decision to publish.

Competing Interests: JN declared receiving a small grant from the Cochrane Collaboration to undertake a component of this this work and received travel expenses to attend meetings from the WHO Alliance for Health Policy and Systems Research and the Norwegian Knowledge Centre for the Health Services. All other authors have declared that no competing interests exist.

Abbreviations: CERQual, Confidence in the Evidence from Reviews of Qualitative research; GRADE, Grading of Recommendations Assessment, Development, and Evaluation; WHO, World Health Organization.

Provenance: Not commissioned; externally peer reviewed

Introduction

The systematic use of research evidence to inform health and social policies is becoming more common among governments, international organisations, and other health institutions, and systematic reviews of intervention effectiveness are now used frequently to inform policy decisions. However, evidence of effectiveness is not sufficient to inform decisions on health and social interventions. Decision makers also need information on the feasibility and acceptability of interventions, so as to better understand factors that may influence their implementation [1,2]. Evidence informing the implementation of an intervention within a health or social care system may be obtained from a range of research, including qualitative research. Furthermore, there has been a rapid increase in the number of syntheses of qualitative research being undertaken and in the development of new methods in this area [3–5].

Most systematic reviewers of qualitative research evidence agree that there is a need to distinguish good quality primary studies from those of poor quality and, further, that structured approaches are needed to enhance the consistency and transparency of any approach taken [6]. While this may give an indication of the trustworthiness of individual studies, and of the review's evidence base as a whole, it does not inform the decision maker about individual findings within a review, which will be produced through the synthesis of different combinations of findings from studies in the review. Typically, policy makers and other end users use these individual findings (Box 1) to inform decisions about health or social care interventions. We therefore need an approach for assessing how much confidence to place in specific review findings to help users judge how much emphasis they should give to these findings in their decisions.

For findings from systematic reviews of the effectiveness of interventions, the Grading of Recommendations Assessment, Development, and Evaluation (GRADE) approach is now in common use. GRADE allows a consistent and transparent assessment of confidence in evidence of effectiveness for each outcome considered in a systematic review. Key elements in a GRADE assessment, applied to each review outcome, include the risk of bias in the included studies, the relevance or directness of these studies to the review question, the consistency of results from these studies, the precision of the estimate, and the risk of publication bias in the contributing evidence. Such assessments of findings from reviews of effectiveness are a critical component of developing recommendations on health care interventions [10].

Guideline development groups, and other users of evidence from systematic reviews, are often familiar with the GRADE approach for assessing how much certainty to place in findings from reviews of the effectiveness of interventions. However, GRADE is not appropriate for qualitative evidence. As the demand for syntheses of qualitative evidence increases, so does the need to be able to assess how much confidence to place in findings from these syntheses [1]. At present, there is no established approach for indicating how confident we can be in the findings from qualitative evidence syntheses, although one previous study attempted to adapt the GRADE approach to qualitative review findings in a mixed-methods synthesis [11], while another study described a tool developed specifically to assess confidence in findings for meta-aggregative qualitative evidence syntheses [12]. The lack of an established approach is an important constraint to incorporating qualitative evidence on the acceptability and feasibility of health interventions into tools to support decision making, including the GRADE Evidence to Decision frameworks [13]. This paper describes a new approach for assessing how much confidence to place in findings from qualitative evidence syntheses.

Box 1. What Is a Review Finding?

The CERQual approach is applied to individual review findings from a qualitative evidence synthesis. Critical to the application and development of CERQual is, therefore, an understanding of what a review finding is. While it may be obvious in some syntheses, for others it will be unclear to which findings (or at which level of synthesis) the CERQual approach should be applied.

For the purposes of CERQual, we define a review finding as an analytic output from a qualitative evidence synthesis that, based on data from primary studies, describes a phenomenon or an aspect of a phenomenon.

By "phenomenon," we mean the issue that is the focus of the qualitative inquiry. The phenomenon of interest may be a health or social intervention or issue (S1 Table).

How review findings are defined and presented depends on many factors, including the review question, the synthesis methods used, the intended purpose or audience of the synthesis, and the richness of the data available. The large number of approaches to qualitative synthesis range, in terms of purpose, from those that aim to identify and describe major themes to those that seek more generalizable, interpretive explanations that can be used for theory building [7]. Furthermore, many syntheses use both of these approaches or include findings that cannot clearly be defined as either descriptive or interpretive.

An example of a qualitative evidence synthesis that presents different levels of findings is that by Thomas and colleagues on barriers to healthy eating among children. At a more descriptive level, the review includes the finding that children's food choices are constrained by the availability of food for school dinners and by pressures to choose and eat food quickly. At a more interpretive level, the review attempts to build theory around children's eating habits. The review authors discuss the finding that children did not see it as their role to be interested in health, preferring to prioritize taste, and that buying healthy food was not a legitimate use of their own pocket money [8]. Similarly, a recent synthesis on factors affecting the implementation of lay health worker programmes presented a range of more descriptive findings tied to programme acceptability among different stakeholders, lay health worker motivation, and health systems constraints. The review authors organised these findings in a logic model in which they proposed different chains of events in which specific lay health worker programme components led to particular intermediate or long-term outcomes, and in which specific moderators positively or negatively affected this process [9].

To date, CERQual has been applied to more descriptive-level review findings in syntheses that have been commissioned and used for guideline development for health systems. Given the range of synthesis methods available and the many options for presenting review findings, review authors will need to judge on a case-by-case basis when it is appropriate to apply the CERQual approach. As experience in applying the approach is gained, guidance will be developed on the range of review findings to which CERQual can be applied.

Methods

Development of the Confidence in the Evidence from Reviews of Qualitative research (CERQual) Approach

The CERQual approach was initially developed in 2010 to support a panel that was using qualitative evidence syntheses to develop a new World Health Organization (WHO) guideline [14].

The technical team for this guideline needed an approach for consistently and transparently assessing and presenting any concerns about the qualitative evidence synthesis findings being used by the panel to inform the guideline.

To develop CERQual, we established a working group of researchers involved in undertaking evidence syntheses. We needed an approach that could be applied to findings from common types of qualitative study designs (e.g., ethnography, case studies) and data (e.g., from interviews, observational), was easy to use, provided a systematic approach to making judgements, allowed these judgements to be reported transparently, and allowed judgements to be understood easily, including by readers without an in-depth understanding of qualitative methods. This work was informed by both the principles of qualitative research and the principles used to develop GRADE for effectiveness [15]. The guidance in this paper has also been developed in collaboration and agreement with the GRADE Working Group (www.gradeworkinggroup.org).

CERQual was developed iteratively. Our first version included two components—methodological limitations and coherence—and was piloted on five syntheses [9,16–19]. In 2013, we presented the CERQual approach to researchers, methodologists, and decision makers at a number of events, including the Cochrane Colloquium [20] and a GRADE Working Group meeting. We then revised the approach, based on feedback from these sessions, to include an additional two components. This gave the approach a total of four components: methodological limitations, relevance, coherence, and adequacy of data. We also identified a further potential component—dissemination bias—as being important but requiring further methodological research before we are able to make a decision on whether this should be included in the CERQual approach (Box 2).

To obtain further feedback, we presented the current, four-component version of the approach in 2014 to a group of 25 invited methodologists, researchers, and end users from more than twelve international organizations, with a broad range of experience in qualitative research, the development of GRADE, or guideline development.

Our work is not attempting to produce a rigid checklist to appraise review findings—the risks of applying such critical appraisal checklists unreflectively to qualitative primary studies have been discussed widely in the literature [6,22–24]. Rather, CERQual is conceived of as a structured approach to appraisal that requires reviewer judgement and interpretation

Box 2. Dissemination Bias in Qualitative Research

Dissemination bias (also referred to as publication bias) may be important for qualitative evidence syntheses in situations in which selective dissemination of qualitative studies or the findings of qualitative studies results in systematic distortion of the phenomenon of interest (see S1 Table). However, empirical evidence on the extent of dissemination bias in qualitative research is very limited—to our knowledge, only one small study on this issue has been conducted [21]. Further, empirical evidence of the impacts of dissemination bias on qualitative evidence syntheses does not, to our knowledge, exist at present. We also do not have methods available for exploring whether the findings of a synthesis have been distorted systematically by dissemination bias.

A programme of methodological work is currently underway to explore both the extent and nature of dissemination bias in qualitative research and how such bias impacts on qualitative evidence synthesis findings.

throughout the approach. Our reasons for developing it are both epistemological and pragmatic. We believe that we should have different degrees of confidence in different findings from a qualitative evidence synthesis because of differences in the evidence that inform each finding. In developing the CERQual components, we have strived to capture core concerns of qualitative researchers such as richness of findings and the explanatory power of any interpretive concepts. We have also tried to respond to the needs of decision makers and other users for research that can usefully inform their policy and practice questions. Without a structured approach, judgements about confidence in a finding are likely to be made anyway by users, but in an ad hoc fashion. Indeed, without a structure for thinking about confidence in findings of qualitative evidence syntheses, these projects risk being further marginalised and underused in informing policy practice. We anticipate that the approach may be refined in the future through further development by the CERQual team and through experience in using the approach. The four CERQual components are described in detail below.

Results

Purpose of CERQual

CERQual aims to transparently assess and describe how much confidence decision makers and other users can place in individual review findings from syntheses of qualitative evidence. We have defined confidence in the evidence as an assessment of the extent to which the review finding is a reasonable representation of the phenomenon of interest. Put another way, it communicates the extent to which the research finding is likely to be substantially different from the phenomenon of interest. By substantially different, we mean different enough that it might change how the finding influences a practical or policy decision about health, social care, or other interventions.

A CERQual assessment provides decision makers with the information they need to decide how much emphasis to give to a particular review finding. Box 1 outlines how a review finding is defined for the purpose of CERQual assessments, Box 3 summarises the purpose of CERQual

Box 3. The Purpose of CERQual and What CERQual Is Not Intended to Address

The CERQual approach transparently assesses and describes how much confidence to place in individual review findings from syntheses of qualitative evidence.
CERQual is not intended for the following:

- Critical appraisal of the methodological limitations of an individual qualitative study
- Critical appraisal of the methodological limitations of a qualitative evidence synthesis
- Appraisal of quantitative or mixed methods data
- Assessing how much confidence to place in the findings from what are sometimes described as "narrative" or "qualitative" summaries of the effectiveness of an intervention, in systematic reviews of effectiveness in which meta-analysis is not possible
- Assessing how much confidence to place in the overall findings of a qualitative evidence synthesis. Rather, it focuses on assessing how much confidence to place in individual review findings from qualitative evidence syntheses

as well as specifying the issues that CERQual is not intended to address, and S1 Table describes other definitions relevant to CERQual.

Components of CERQual

Four components contribute to an assessment of confidence in the evidence for an individual review finding: methodological limitations, relevance, coherence, and adequacy of data (Table 1). Concerns about any of these components may lower our confidence in a review finding. Each component is discussed in more detail below. The CERQual components reflect similar concerns to the elements included in the GRADE approach for assessing the certainty of evidence on the effectiveness of interventions (S2 Table). However, CERQual considers these issues from a qualitative perspective. This paper focuses on situations in which review authors assess how much confidence to place in findings from a review they have undertaken themselves. It may also be possible to apply CERQual to the findings from qualitative evidence syntheses performed by others, and this is discussed later.

Methodological Limitations

Definition and explanation. Methodological limitations are the extent to which there are problems in the design or conduct of the primary studies that contributed evidence to a review finding. When the primary studies underlying a review finding are shown to have important methodological limitations, we are less confident that the review finding reflects the phenomenon of interest.

Operationalizing "methodological limitations". When undertaking a qualitative evidence synthesis, review authors should assess the methodological limitations of each primary study included in the synthesis. This should be done using a relevant checklist or tool (for instance, [25–27]). An assessment of methodological limitations should be based on the methodological strengths and weaknesses of each study as there is no hierarchy of study design within qualitative research. Review authors should present and explain these assessments in the review appendices.

When assessing the methodological limitations of the evidence underlying a review finding, review authors must make an overall judgement based on all of the primary studies contributing to the finding. This judgement needs to take into account each study's relative contribution to the evidence, the types of methodological limitations identified, and how those methodological limitations may impact on the specific finding.

How a primary study was conducted may constitute a methodological limitation for one review finding but not for another finding. For instance, in a study on sexual behaviour among teenagers, a decision to use focus group discussions to collect data may be regarded as a

Table 1. Components of the CERQual approach.

Component	Definition
Methodological limitations	The extent to which there are problems in the design or conduct of the primary studies that contributed evidence to a review finding
Relevance	The extent to which the body of evidence from the primary studies supporting a review finding is applicable to the context (perspective or population, phenomenon of interest, setting) specified in the review question
Coherence	The extent to which the review finding is well grounded in data from the contributing primary studies and provides a convincing explanation for the patterns found in these data
Adequacy of data	An overall determination of the degree of richness and quantity of data supporting a review finding

doi:10.1371/journal.pmed.1001895.t001

limitation for findings about teenagers' perceptions of risky or illegal behaviour but may not be regarded as a problem for findings about teenagers' perceptions of sex education. This is because teenagers may be less willing to talk frankly about the former within a group setting.

Implications when methodological limitations are identified. When we identify methodological limitations for a particular review finding, this may indicate that primary researchers in this area need to use more appropriate methods or to report the methods used more clearly in future studies.

Relevance

Definition and explanation. Relevance is the extent to which the body of evidence from the primary studies supporting a review finding is applicable to the context specified in the review question. This may relate to, for example, the perspective or population researched, the phenomenon of interest or the setting.

Relevance is important in assessing confidence as it indicates to the end user the extent to which the contexts of the primary studies contributing evidence to a finding are aligned with the context specified in the review question. When the contexts of the primary studies underlying a review finding are substantively different from the context of the review question, we are less confident that the review finding reflects the phenomenon of interest.

Operationalizing "relevance". For the most part, a review's inclusion criteria for studies are aligned with the review question, and the included studies are therefore relevant to the review question. However, there are situations in which studies are of reduced relevance. This can be due to differences relating to any of the main domains in a typical review question. This may include differences in the perspective or population, the phenomenon of interest or intervention, the setting, or the time frame. We propose three ways in which relevance could be categorized: indirect relevance, partial relevance, and uncertain relevance.

The evidence supporting a review finding may be indirectly relevant if one of the review domains above, such as perspective or setting, has been substituted with another. For example, the authors of a qualitative evidence synthesis plan to address the question of people's responses to the swine flu pandemic, but they find no studies exploring this question. However, the review authors identify studies looking at people's responses to the bird flu pandemic. These studies are included as a likely alternative indicator of people's responses to the phenomenon of interest (swine flu). Indirect relevance implies that the review authors (or others) have made assumptions about the relevance of the findings to the original review question.

Relevance may be partial when the studies identified for a review address only a subset of the relevant review domains above. For example, in a synthesis exploring how children living in care institutions across Europe experience different models of care, the review authors only identify studies from Norway. Only part of the review question is therefore addressed. Partial relevance implies that the review question is only addressed in a limited way. When this occurs, review authors need to determine which domains in the review question are most important in assessing relevance.

The degree of relevance may be assessed as uncertain when the review authors are unsure about the extent to which the focus of the included studies reflects the phenomenon of interest because of deficiencies in the reported details of the population, intervention, or settings. For example, in a qualitative evidence synthesis exploring cancer patients' experience with mindfulness-based training, the review authors identify several studies. However, it is unclear whether all of these training programmes include similar approaches to both mindfulness and mindfulness-based training. Uncertain relevance implies that it is difficult to draw conclusions about the relevance of the review finding to the review question.

Our confidence in a review finding may be weakened if the relationship between the contexts of the primary studies and the review question is indirect, partial, or uncertain. Review authors should describe any concerns regarding the extent to which the review finding reflects the context of interest. This will allow end users to better understand the assessment and consider the finding in relation to their own context.

Implications when concerns regarding relevance are identified. Concerns regarding relevance could indicate a need for more research in different contexts and for better reported primary research. However, they could equally indicate that the phenomenon that is the focus of the review is not prevalent in a given context. For example, a review of parental worries about their children's health may not uncover European-based studies in which dysentery is mentioned. Rather than indicating gaps in relevant data, this is more likely to be because parents in Europe do not discuss fear of dysentery when asked specifically about their children's health since it is not a common health problem in most high-income settings.

Coherence

Definition and explanation. Qualitative review findings are developed by identifying patterns in the data across the primary studies included in an evidence synthesis. The coherence of the review finding addresses the question of whether the finding is well grounded in data from these primary studies and provides a convincing explanation for the patterns found in these data.

Coherence in the data contributing to a review finding may be contextual, where patterns are found across studies that are similar to each other with respect to population, interventions, or settings; or conceptual, where patterns in the data from the underlying studies can be explained in relation to new or existing theory. Patterns need to be explained and supported through data presented in the primary studies or through hypotheses developed by the primary study authors or the review authors.

Review findings are sometimes challenged by outlying, contrasting, or even disconfirming data from the primary studies that do not support or that directly challenge the main finding. Review authors should look actively for such data that complicate or challenge their main findings [28] and attempt to explain these variations or exceptions. When there is no convincing explanation for these variations or exceptions, we are less confident that the review finding reflects the phenomenon of interest. Guidance on what constitutes a convincing explanation needs further development.

Operationalizing "coherence". Confidence in a review finding may be lower when there is an unexplained lack of coherence. When theories or explanations are used to explain similarities or variations, review authors should specify whether the theory or explanation is internally generated (i.e., the theory or explanation comes from one or several of the studies underlying the review finding), externally sourced (i.e., the theory or explanation is imported from an external source, such as an established concept or theory), or original (i.e., the theory or explanation has been developed by the review authors as part of the synthesis process).

Reasons why it may be difficult to explain the variation in data across individual studies contributing to a finding include that the available data are too thin [29], outlying or disconfirming cases are not well explored, the review authors do not know the field sufficiently well to generate an explanation, the theory used to inform the review is incomplete or flawed, or the study sampling for the review was limited. Study sampling and the extent to which outlying cases were explored may also be assessed as part of the "methodological limitations" component of CERQual.

Since the patterns that constitute a review finding are created by the review authors, assessing coherence during the synthesis offers an opportunity for "self-check" or reflection. Examining the coherence of the review findings gives review authors an opportunity to reflect on the

extent to which the pattern captured in the review finding really is contextually or conceptually coherent. It also gives review authors an opportunity to offer a convincing explanation for the patterns they have found and to note the presence of disconfirming cases.

Implications when concerns about coherence are identified. Concerns regarding the coherence of a review finding can have several implications: firstly, review authors should consider using the patterns found across primary studies to generate new hypotheses or theory regarding the issue addressed by the finding. Secondly, a lack of coherence in relation to a particular review finding may suggest that more primary research needs to be done in that area and that the review should perhaps be updated once those data are available. Finally, when a review has used a sampling procedure to select studies for inclusion in the review [30], future updates of the review could reconfigure the sampling to explore the variation found.

Adequacy of Data

Definition and explanation. Adequacy of data is an overall determination of the degree of richness and quantity of data supporting a review finding.

In assessing adequacy of data, we define "rich data" as data that provide us with sufficient detail to gain an understanding of the phenomenon we are studying—for instance, an understanding of participants' perceptions and experiences of a given topic. In contrast, thin data do not provide enough detail to develop an understanding of the phenomenon of interest.

In addition to data richness, quantity of data is also important. When a review finding is supported by data from only one or few primary studies, participants, or observations, we are less confident that the review finding reflects the phenomenon of interest. This is because when only a few studies or only small studies exist or when few are sampled, we do not know whether studies undertaken in other settings or groups would have reported similar findings.

Operationalizing "adequacy of data". Confidence in a review finding may be lower when there are concerns regarding whether there are adequate amounts of data contributing to a review finding. This could include concerns about the richness of the data or the number of studies, participants, or observations from which the data are drawn.

Review authors need to judge adequacy in relation to the claims made in a specific review finding. There are therefore no fixed rules on what constitutes sufficiently rich data or an adequate quantity of data. When considering whether there are adequate data, review authors may find the principle of saturation of data useful or could consider the extent to which additional data are likely to change the finding [31–34]. Review authors should also look for disconfirming cases. More work is needed on how to apply these strategies in the context of a qualitative evidence synthesis.

Implications when concerns regarding the adequacy of data are identified. When adequacy of data is not achieved, this may suggest that more primary research needs to be done in relation to the issue discussed in the review finding and that the review should be updated once that research is available. Inadequate data may indicate that the review question was too narrow and that future syntheses should consider a broader scope or include primary studies that examine phenomena that are similar, but not identical, to that under consideration in the synthesis. This, in turn, might have implications for assessment of relevance.

Making an Assessment of Level of Confidence for a Finding

As noted earlier, our confidence in the evidence is an assessment of the extent to which the review finding is a reasonable representation of the phenomenon of interest (S1 Table). This assessment is based on the judgements made for each of the four CERQual components. These judgements can be summarised in a CERQual Qualitative Evidence Profile (Table 2). While

Table 2. Example of a CERQual Qualitative Evidence Profile*[#].

Objective: To identify, appraise, and synthesise qualitative research evidence on the barriers and facilitators to the implementation of lay health worker programmes for maternal and child health[#]

Perspective: Experiences and attitudes of stakeholders about lay health worker programmes in any country

Included programmes: Programmes that were delivered in a primary or community health care setting, that intend to improve maternal or child health, and that had used any type of lay health worker, including community health workers, village health workers, birth attendants, peer counsellors, nutrition workers, and home visitors

Review Finding	Studies Contributing to the Review Finding	Assessment of Methodological Limitations	Assessment of Relevance	Assessment of Coherence	Assessment of Adequacy	Overall CERQual Assessment of Confidence	Explanation of Judgement
While regular salaries were not part of many programmes, other monetary and nonmonetary incentives, including payment to cover out-of-pocket expenses and "work tools" such as bicycles, uniforms, or identity badges, were greatly appreciated by lay health workers.	Studies 2; 5; 11; 12; 22; 29	Minor methodological limitations (five studies with minor and one study with moderate methodological limitations)	Minor concerns about relevance (studies of lay health worker programmes from five countries and three continents: United States, Uganda, Nepal, Kenya, and India)	Minor concerns about coherence (data reasonably consistent within and across all studies)	Minor concerns about adequacy (six studies that together offered moderately rich data overall)	**Moderate confidence**	This finding was graded as moderate confidence because of minor concerns regarding methodological limitations, relevance, coherence, and adequacy.
Some unsalaried lay health workers expressed a strong wish for regular payment.	Studies 5; 13	Minor methodological limitations (both studies had minor methodological limitations)	Moderate concerns about relevance (partial relevance, as the studies were from only two settings, both of which were in Africa)	Minor concerns about coherence (data consistent within and across both studies)	Substantial concerns about adequacy (only two studies, both offering thin data)	**Low confidence**	This finding was graded as low confidence because of moderate concerns regarding relevance and substantial concerns regarding adequacy of data.

* Findings were taken from [9] and adapted to fit the context of this article.

[#] The synthesis findings presented here are drawn from the wider thematic synthesis undertaken for this review. The themes identified were summarised into evidence statements, as illustrated in this table. The methods are described in more detail in [9].

doi:10.1371/journal.pmed.1001895.t002

each CERQual component should initially be assessed individually, review authors also need to look iteratively across the components in order to make a final assessment as components may interact, as noted above, and also to avoid "double downgrading" for the same issue.

To indicate our assessment of confidence, we propose four levels: high, moderate, low, or very low. This is a similar approach to that used in the GRADE tool for assessing confidence in the evidence on the effectiveness of interventions [35]. The levels of confidence for CERQual are defined in Table 3. We propose that all review findings start off as "high confidence" and are then "rated down" by one or more levels if there are concerns regarding any of the CERQual components. This starting point of "high confidence" reflects a view that each review finding should be seen as a reasonable representation of the phenomenon of interest unless there

Table 3. The CERQual approach—Definitions of levels of confidence in a review finding.

Level	Definition
High confidence	It is highly likely that the review finding is a reasonable representation of the phenomenon of interest
Moderate confidence	It is likely that the review finding is a reasonable representation of the phenomenon of interest
Low confidence	It is possible that the review finding is a reasonable representation of the phenomenon of interest
Very low confidence	It is not clear whether the review finding is a reasonable representation of the phenomenon of interest.

doi:10.1371/journal.pmed.1001895.t003

are factors that would weaken this assumption. Confidence should be assessed for each review finding individually and not for the review as a whole. Future papers will describe in more detail for each CERQual component the circumstances under which confidence in a review finding should be rated down.

The assessment of confidence for a review finding is a judgement, and it is therefore particularly important to include an explanation of how this judgement was made. This is discussed further below. Our experience to date in applying CERQual suggests that it may be difficult to achieve "high confidence" for review findings in many areas, as the underlying studies often reveal methodological limitations or there are concerns regarding the adequacy of the data. Those assessing confidence in review findings should specify as far as possible how future studies could address the concerns identified.

Using a "Summary of Qualitative Findings Table" to Summarise the Judgements Made Using CERQual

A summary of qualitative findings table can be used to summarise the key findings from a qualitative evidence synthesis and the confidence in the evidence for each of these findings, as assessed using the CERQual approach. The table should also provide an explanation of the CERQual assessments. An example of a summary of qualitative findings table is provided in Table 4. There are several advantages to providing a succinct summary of each review finding and an explanation of the CERQual assessment for that finding. Firstly, this may encourage review authors to consider carefully what constitutes a finding in the context of their review and to express these findings clearly (Box 1). Secondly, these tables may facilitate the uptake of qualitative evidence synthesis findings into decision making processes, for example, through evidence-to-decision frameworks [13]. Thirdly, these tables help to ensure that the judgements underlying CERQual assessments are as transparent as possible.

Applying the CERQual Approach

The first version of the CERQual approach has been applied in five reviews [9,16–19], three of which were used by WHO as the basis for the development of a global guideline [14]. The current version of CERQual has been used in one published review [36] and is currently being used in a further ten reviews, at least half of which are being produced to support WHO guidance. This experience has highlighted a number of factors that review authors should consider when applying CERQual to review findings, and we discuss these factors below.

General considerations. To date, the application of CERQual to each review finding has been through discussions among at least two review authors. This seems preferable to use by a single reviewer as it offers an opportunity to discuss judgements and may assist review authors in clearly describing the rationale behind each assessment. In addition, multiple reviewers from

Table 4. Example of a CERQual Summary of Qualitative Findings table*#.

Review Finding	CERQual Assessment of Confidence in the Evidence	Explanation of CERQual Assessment	Studies Contributing to the Review Finding
**Objective:** To identify, appraise, and synthesise qualitative research evidence on the barriers and facilitators to the implementation of lay health worker programmes for maternal and child health#			
**Perspective:** Experiences and attitudes of stakeholders about lay health worker programmes in any country			
**Included programmes:** Programmes that were delivered in a primary or community health care setting, that intend to improve maternal or child health, and that had used any type of lay health worker, including community health workers, village health workers, birth attendants, peer counsellors, nutrition workers, and home visitors			
While regular salaries were not part of many programmes, other monetary and nonmonetary incentives, including payment to cover out-of-pocket expenses and "work tools" such as bicycles, uniforms, or identity badges, were greatly appreciated by lay health workers.	Moderate	This finding was graded as moderate confidence because of minor concerns regarding methodological limitations, relevance, coherence, and adequacy.	Studies 2; 5; 11; 12; 22; 29
Some unsalaried lay health workers expressed a strong wish for regular payment.	Low	This finding was graded as low confidence because of moderate concerns regarding relevance and substantial concerns regarding adequacy of data.	Studies 5; 13

*Findings were taken from [9] and adapted to fit the context of this article.

The synthesis findings presented here are drawn from the wider thematic synthesis undertaken for this review. The themes identified were summarised into evidence statements, as illustrated in this table. The methods are described in more detail in [9], and the CERQual assessments for each component are shown in Table 2.

doi:10.1371/journal.pmed.1001895.t004

different disciplinary backgrounds may offer alternative interpretations of confidence—an approach that has also been suggested to enhance data synthesis itself [28]. The approach is intended to be applied by review authors with experience in both primary qualitative research and qualitative evidence synthesis.

Assessments of each CERQual component are based on judgements by the review authors, and these judgements need to be described clearly and in detail. Providing a justification for each assessment, preferably in a summary of qualitative findings table, is important for the end user, as this shows how the final assessment was reached and increases the transparency of the process. Further, when end users are seeking evidence for a question that differs slightly from the original review question, they are able to see clearly how the assessment of confidence has been made and to adjust their own confidence in the review finding accordingly.

When making judgements using the CERQual approach, review authors need to be aware of the interactions between the four components. At this stage, CERQual gives equal weight to each component, as we view the components as equally important. Further research is needed on whether equal weighting is appropriate and on areas in which there may be overlap between components.

Our experience applying the CERQual approach so far has indicated that it is easiest to begin with an assessment of methodological limitations. Thereafter, it does not seem to be important in which order the other three components are assessed, as the process is iterative.

It is probably most appropriate for review authors to apply the CERQual approach to their own review, given that prior familiarity with the evidence is needed in order to make reasonable judgements concerning methodological limitations, coherence, relevance, and adequacy of data. However, in principle the approach could be applied to review findings from well-conducted reviews by people other than the review authors. Guidance for this will be developed in the future.

Considerations when assessing methodological limitations. Qualitative research encompasses a wide range of study designs, and there are multiple tools and approaches for assessing the strengths and weaknesses of qualitative studies [26,27,37–40]. It is currently not possible to recommend a widely agreed upon, simple, and easy to use set of criteria for assessing methodological limitations for the many types of qualitative studies, and this may not be desirable given continued debates regarding different approaches and our desire for the CERQual approach to be used by the range of qualitative researchers involved in evidence synthesis. However, we believe that it is important to try to identify a minimum set of "core domains" for assessing methodological limitations, and this is a key area for future research.

Considerations when assessing relevance. In the application of CERQual to date, relevance has been assessed by review authors and not by users, such as decision makers and those who support them or consumer groups. There may be instances in which such users would like to use review findings from a relevant synthesis, but their context differs to some extent from that specified in the review question. Transparent reporting of the assessment of relevance by the review authors provides these users with a starting point from which to understand the reasons behind the assessment. However, it may be difficult for users who are not familiar with the primary studies to assess the relevance to their own context.

Considerations when assessing coherence. With the CERQual assessment in mind, review authors may be tempted to "smooth out" review findings to eliminate variation or to formulate review findings vaguely in order to artificially increase coherence. However, it is not the intention of CERQual to reduce variation within review findings. Identifying both similarities and differences in the primary data, including accounting for disconfirming cases, is an important part of developing review findings. Review authors should not attempt to create findings that appear more coherent through ignoring or minimising important disconfirming cases. As Patton (1999) points out, "Where patterns and trends have been identified, our understanding of those patterns and trends is increased by considering the instances and cases that do not fit within the pattern" ([41] p. 1191). Moreover, users of qualitative evidence syntheses are often specifically interested in where a review finding is not relevant or applicable, so as to avoid implementing interventions or guidelines that may be inappropriate or not feasible in their specific context.

Considerations when assessing adequacy of data. While numbers can be important and useful in qualitative research, qualitative analysis generally focuses on text-based data [42]. The CERQual component of adequacy of data is not intended to encourage the counting of numbers of studies contributing to a review finding, but rather to focus review authors' attention on where data may be thin or limited in relation to a review finding. In addition, fewer, more conceptually rich studies contributing to a finding may be more powerful than a larger number of thin, descriptive studies.

Discussion

CERQual provides users of evidence with a systematic and transparent assessment of how much confidence can be placed in individual review findings from syntheses of qualitative evidence. In addition, the use of CERQual could help review authors to consider, analyse, and report review findings in a more useful and usable way. Qualitative evidence syntheses share with primary qualitative data analysis the need for multiple rounds of revisiting the data "as additional questions emerge, new connections are unearthed, and more complex formulations develop along with a deepening understanding of the material" [43]. The CERQual approach offers review authors a further opportunity for a more structured approach to analysing data. It guides them through a process of examining and appraising the methodological limitations,

Box 4. Way Forward and Research Agenda for CERQual

CERQual is a work in progress, and the following steps are planned to further develop the approach:

- Detailed guidance for review authors and others who wish to apply the approach is currently being developed. This guidance will address each component of CERQual, describe the approach to assessing levels of confidence, outline how to develop summary of qualitative findings tables, and provide worked examples.
- To date, CERQual has been piloted on evidence syntheses that have used framework [44] or narrative synthesis approaches [45] and that have produced largely descriptive findings. The approach now needs to be tested on syntheses that use other methods or that attempt to develop more explanatory findings such as midlevel theory generation, logic models, or conceptual frameworks. Plans for this are currently underway. This testing will help both to assess whether the approach needs to be expanded or adapted to accommodate different types of findings from the wide range of review approaches currently in use [46] and to develop appropriate guidance for this.
- Given the range of synthesis methods available and the many options for presenting review findings, review authors will need to judge on a case-by-case basis when it is appropriate to apply CERQual. Developing guidance on this is also an important area for further methodological research.
- The development of CERQual has identified several priority issues for methodological research, including identifying core domains for the assessment of methodological limitations in primary qualitative studies and exploring how to apply these, investigating the most appropriate order in which to apply the CERQual components to a finding, understanding the role of "dissemination bias" (e.g., whether studies with "novel" findings are more likely to be published) in the context of qualitative research, and exploring the circumstances under which it may be appropriate to increase or "rate up" confidence in a review finding in relation to a CERQual component.
- Sampling approaches may be employed in qualitative evidence synthesis as part of a priori inclusion criteria (e.g., based on language or study design) or later in the review process after all potentially relevant studies are identified. Studies may be sampled based on, for instance, principles of data saturation or theoretical sampling, or methodological quality [30]. Experience is needed with these types of reviews in order to establish the degree to which sampling impacts on CERQual assessments.

relevance, coherence, and adequacy of the data contributing to a review finding. The development of CERQual has identified a number of important research questions, and these are summarised in Box 4.

Some methodologists have critiqued tools that propose explicit criteria for appraising the quality of qualitative research, questioning whether such tools can adequately assess "quality" for this research method [22]. We take the standpoint, however, that ways of appraising both primary and secondary qualitative research are needed. Such approaches need to be appropriate to, and take into account the diversity of, qualitative methods [27,37,39]. As noted above, users of both primary qualitative research findings and qualitative evidence synthesis findings routinely make these judgements when reading and using these types of research. However, the

judgements made by these users are implicit, which makes it difficult for others to understand and critique them—an important limitation when findings from such research are then used to inform decisions about health and social policies. CERQual attempts to make assessments of confidence in the evidence more systematic and transparent while accepting that these assessments are judgements that are likely to vary across assessors.

An intended consequence of the CERQual approach is to improve methodological quality and reporting standards in primary qualitative research. For an adequate CERQual assessment to be made, the authors of primary studies need to provide sufficient information about the methods they have used. Wide use of CERQual may thus encourage more thorough reporting of qualitative research methods.

To support the further development of CERQual and facilitate wide involvement of methodologists, researchers, reviewers, and other stakeholders in this process, we have established a GRADE-CERQual Project Group (see: www.cerqual.org). This is an informal collaboration of people with an interest in how to assess confidence in evidence from qualitative evidence syntheses and is a subgroup of the GRADE Working Group. We would encourage those with an interest in this area to join the group and contribute to the development of the CERQual approach.

Supporting Information

S1 Table. Key definitions relevant to CERQual.
(PDF)

S2 Table. Comparison of the CERQual components and the elements of GRADE.
(PDF)

S1 Alternative Language Summary Points. French translation of the Summary Points.
(PDF)

S2 Alternative Language Summary Points. Italian translation of the Summary Points.
(PDF)

S3 Alternative Language Summary Points. Norwegian translation of the Summary Points.
(PDF)

S4 Alternative Language Summary Points. Spanish translation of the Summary Points.
(PDF)

Acknowledgments

Our thanks for their feedback to those who participated in the first GRADE-CERQual Project Group meeting in Barcelona in January 2014: Elie Akl, Zhenggang Bai, Rigmor Berg, Meghan Bohren, Jackie Chandler, Karen Daniels, Bela Ganatra, Andy Oxman, Tomas Pantoja, Kent Ranson, Rebecca Rees, Holger Schünemann, Birte Snilstveit, James Thomas, Hilary Thompson, and Josh Vogel. In addition, we received valuable feedback on this manuscript from Paul Elias Alexander, Meghan Bohren, Philippe du Clos, Joerg Meerpohl, Jasvinder Singh, and Özge Tuncalp and from discussions at several meetings of the GRADE Working Group.

Author Contributions

Wrote the first draft of the manuscript: SL CG HMK. Contributed to the writing of the manuscript: SL CG HMK BC CJC MG JN AB RG AR. Agree with the manuscript's results and

conclusions: SL CG HMK BC CJC MG JN AB RG AR. All authors have read, and confirm that they meet, ICMJE criteria for authorship.

References

1. Lewin S, Bosch-Capblanch X, Oliver S, Akl EA, Vist GE, et al. (2012) Guidance for Evidence-Informed Policies about Health Systems: Assessing How Much Confidence to Place in the Research Evidence. PLoS Med 9: e1001187. doi: 10.1371/journal.pmed.1001187 PMID: 22448147
2. Oxman AD, Lavis JN, Lewin S, Fretheim A (2009) SUPPORT Tools for evidence-informed health Policymaking (STP) 1: What is evidence-informed policymaking? Health Res Policy Syst 7 Suppl 1: S1. doi: 10.1186/1478-4505-7-S1-S1 PMID: 20018099
3. Gulmezoglu AM, Chandler J, Shepperd S, Pantoja T (2013) Reviews of qualitative evidence: a new milestone for Cochrane. Cochrane Database Syst Rev 11: ED000073. doi: org/10.1002/14651858.ED000073 PMID: 24524152
4. Noyes J, Gough D, Lewin S, Mayhew A, Michie S, et al. (2013) A research and development agenda for systematic reviews that ask complex questions about complex interventions. Journal of Clinical Epidemiology 66: 1262–1270. doi: 10.1016/j.jclinepi.2013.07.003 PMID: 23953084
5. Petticrew M, Rehfuess E, Noyes J, Higgins JPT, Mayhew A, et al. (2013) Synthesizing evidence on complex interventions: how meta-analytical, qualitative, and mixed-method approaches can contribute. Journal of Clinical Epidemiology 66: 1230–1243. doi: 10.1016/j.jclinepi.2013.06.005 PMID: 23953082
6. Petticrew M, Roberts H (2006) Systematic Reviews in the Social Sciences: A Practical Guide. Oxford, UK: Wiley-Blackwell.
7. Gough D, Thomas J, Oliver S (2012) Clarifying differences between review designs and methods. Syst Rev 1: 28. doi: 10.1186/2046-4053-1-28 PMID: 22681772
8. Thomas J, Sutcliffe K, Harden A, Oakley A, Oliver S, et al. (2003) Children and healthy eating: a systematic review of barriers and facilitators. London: EPPI-Centre, Social Science Research Unit, Institute of Education, University of London.
9. Glenton C, Colvin CJ, Carlsen B, Swartz A, Lewin S, et al. (2013) Barriers and facilitators to the implementation of lay health worker programmes to improve access to maternal and child health: qualitative evidence synthesis. Cochrane Database Syst Rev 10: CD010414. doi: 10.1002/14651858.CD010414.pub2 PMID: 24101553
10. Guyatt G, Oxman AD, Akl EA, Kunz R, Vist G, et al. (2011) GRADE guidelines: 1. Introduction-GRADE evidence profiles and summary of findings tables. J Clin Epidemiol 64: 383–394. doi: 10.1016/j.jclinepi.2010.04.026 PMID: 21195583
11. Goldsmith MR, Bankhead CR, Austoker J (2007) Synthesising quantitative and qualitative research in evidence-based patient information. J Epidemiol Community Health 61: 262–70. PMID: 17325406
12. Munn Z, Porritt K, Lockwood C, Aromataris E, Pearson A (2014) Establishing confidence in the output of qualitative research synthesis: the ConQual approach. BMC Medical Research Methodology 14: 108. doi: 10.1186/1471-2288-14-108 PMID: 25927294
13. Treweek S, Oxman AD, Alderson P, Bossuyt PM, Brandt L, et al. (2013) Developing and Evaluating Communication Strategies to Support Informed Decisions and Practice Based on Evidence (DECIDE): Protocol and Preliminary Results. Implement Sci 9; 8: 6. doi: 10.1186/1748-5908-8-6 PMID: 23302501
14. WHO (2012) Optimizing health worker roles to improve access to key maternal and newborn health interventions through task shifting. Geneva: World Health Organization.
15. Guyatt GH, Oxman AD, Vist GE, Kunz R, Falck-Ytter Y, et al. (2008) GRADE: an emerging consensus on rating quality of evidence and strength of recommendations. BMJ 336: 924–926. doi: 10.1136/bmj.39489.470347.AD PMID: 18436948
16. Bohren MA, Hunter EC, Munthe-Kaas HM, Souza JP, Vogel JP, et al. (2014) Facilitators and barriers to facility-based delivery in low- and middle-income countries: a qualitative evidence synthesis. Reprod Health 11: 71. doi: 10.1186/1742-4755-11-71 PMID: 25238684
17. Colvin CJ, de Heer J, Winterton L, Mellenkamp M, Glenton C, et al. (2013) A systematic review of qualitative evidence on barriers and facilitators to the implementation of task-shifting in midwifery services. Midwifery 29: 1211–1221. doi: 10.1016/j.midw.2013.05.001 PMID: 23769757
18. Munthe-Kaas HM, Hammerstrøm KT, Kurtze N, Nordlund KR (2013) Effekt av og erfaringer med kontinuitetsfremmende tiltak i barnevernsinstitusjoner. Oslo: Norwegian Knowledge Centre for the Health Services. Available: http://www.kunnskapssenteret.no/publikasjoner/effekt-av-og-erfaringer-med-kontinuitetsfremmende-tiltak-i-barnevernsinstitusjoner

19. Rashidian A, Shakibazadeh E, Karimi- Shahanjarini A, Glenton C, Noyes J, et al. (2013) Barriers and facilitators to the implementation of doctor-nurse substitution strategies in primary care: qualitative evidence synthesis (Protocol). Cochrane Database of Systematic Reviews 2: CD010412.
20. Lewin S, Glenton C, Munthe-Kaas H, al. e (2013) Assessing how much certainty to place in findings from qualitative evidence syntheses: the CerQual approach. Oral presentation, 20th Cochrane Colloquium, Quebec 2013. 20th Cochrane Colloquium. Quebec.
21. Petticrew M, Egan M, Thomson H, Hamilton V, Kunkler R, et al. (2008) Publication bias in qualitative research: what becomes of qualitative research presented at conferences? J Epidemiol Community Health 62: 552–554. doi: 10.1136/jech.2006.059394 PMID: 18477755
22. Barbour RS (2001) Checklists for improving rigour in qualitative research: a case of the tail wagging the dog? BMJ 322: 1115–1117. PMID: 11337448
23. Barbour RS, Barbour M (2003) Evaluating and synthesizing qualitative research: the need to develop a distinctive approach. J Eval Clin Pract 9: 179–186. PMID: 12787181
24. Dixon-Woods M, Fitzpatrick R, Roberts K (2001) Including qualitative research in systematic reviews: opportunities and problems. J Eval Clin Pract 7: 125–133. PMID: 11489038
25. CASP (2011) Qualitative Appraisal Checklist for Qualitative Research. Critical Appraisal Skills Programme. www.casp-uk.net/#!casp-tools-checklists/c18f8
26. Government Chief Social Researcher's Office (2003) Quality in Qualitative Evaluation: A framework for assessing research evidence. United Kingdom: Cabinet Office.
27. Walsh D, Downe S (2006) Appraising the quality of qualitative research. Midwifery 22: 108–119. PMID: 16243416
28. Booth A, Carroll C, Ilott I, Low LL, Cooper K (2013) Desperately seeking dissonance: identifying the disconfirming case in qualitative evidence synthesis. Qual Health Res 23: 126–141. doi: 10.1177/1049732312466295 PMID: 23166156
29. Ponterotto JG (2006) Brief note on the origins, evolution, and meaning of the qualitative research concept “Thick description”. The Qualitative Report 11: 538–549.
30. Suri H (2011) Purposeful sampling in qualitative research synthesis. Qualitative Research Journal 11: 63–75.
31. Bowen GA (2008) Naturalistic inquiry and the saturation concept: a research note. Qualitative research 8: 16.
32. Francis J, Johnston M, Robertson C, Glidewel L, Entwistle V, et al. (2010) What is an adequate sample size? Operationalising data saturation for theory-based interview studies. Psychology & Health 25: 1229–1245.
33. Guest G, Bunce A, Johnson L (2006) How many interviews are enough? An experiment with data saturation and variability. Field methods 18: 59–82.
34. O'Reilly M, Parker N (2013) 'Unsatisfactory Saturation': a critical exploration of the notion of saturated sample sizes in qualitative research. Qualitative Research 13: 190–197.
35. Guyatt G, Oxman AD, Sultan S, Brozek J, Glasziou P, et al. (2013) GRADE guidelines: 11. Making an overall rating of confidence in effect estimates for a single outcome and for all outcomes. J Clin Epidemiol 66: 151–157. doi: 10.1016/j.jclinepi.2012.01.006 PMID: 22542023
36. Bohren MA, Vogel JP, Hunter EC, Lutsiv O, Makh SK, et al. (2015) The Mistreatment of Women during Childbirth in Health Facilities Globally: A Mixed-Methods Systematic Review. PLoS Med 12: e1001847; discussion e1001847. doi: 10.1371/journal.pmed.1001847 PMID: 26126110
37. Dixon-Woods M, Shaw RL, Agarwal S, Smith JA (2004) The problem of appraising qualitative research. Qual Saf Health Care 13: 223–225. PMID: 15175495
38. Dixon-Woods M, Sutton A, Shaw R, Miller T, Smith J, et al. (2007) Appraising qualitative research for inclusion in systematic reviews: a quantitative and qualitative comparison of three methods. J Health Serv Res Policy 12: 42–47. PMID: 17244397
39. Murphy E, Dingwall R, Greatbatch D, Parker S, Watson P (1998) Qualitative research methods in health technology assessment: a review of the literature. Health Technol Assess 2: iii–ix, 1–274. PMID: 9919458
40. Tong A, Sainsbury P, Craig J (2007) Consolidated criteria for reporting qualitative research (COREQ): a 32-item checklist for interviews and focus groups. Int J Qual Health Care 19: 349–357. PMID: 17872937
41. Patton MQ (1999) Enhancing the quality and credibility of qualitative analysis. Health Services Research 35: 1189–1208.
42. Sandelowski M (2001) Real qualitative researchers do not count: The use of numbers in qualitative research. Research in Nursing & Health 24: 230–240.

43. Berkowitz S (1997) Analyzing qualitative data. In: Frechtling J, Sharp L, editors. User-friendly handbook for mixed method evaluations. Arlington, VA: Division of Research, Evaluation and Communication, National Science Foundation.

44. Booth A, Papaioannou D, Sutton A (2012) Systematic Approaches to a Successful Literature Review. London, UK: Sage Publications.

45. Popay J, Roberts H, Sowden A (2006) Guidance on the Conduct of Narrative Synthesis in Systematic Reviews. A Product from the ESRC Methods Programme. Lancaster: Institute of Health Research.

46. Barnett-Page E, Thomas J (2009) Methods for the synthesis of qualitative research: a critical review. BMC Med Res Methodol 9: 59. doi: 10.1186/1471-2288-9-59 PMID: 19671152
